# Crystal Structure and Stereochemistry Study of 2-Substituted Benzoxazole Derivatives

**DOI:** 10.1155/2014/728343

**Published:** 2014-05-13

**Authors:** Ahmed F. Mabied, Elsayed M. Shalaby, Hamdia A. Zayed, Esmat El-Kholy, Ibrahim S. A. Farag, Naima A. Ahmed

**Affiliations:** ^1^Crystallography Laboratory, Physics Division, National Research Center, Dokki, Giza 12622, Egypt; ^2^Physics Department, Women's College, Ain Shams University, Cairo 11757, Egypt

## Abstract

The structure of 2-[(4-chlorophenylazo) cyanomethyl] benzoxazole, C_15_H_9_ClN_4_O (I), has triclinic ($P\overset{®}{1}$) symmetry. The structure displays N–H ⋯ N hydrogen bonding. The structure of 2-[(arylidene) cyanomethyl] benzoxazoles, C_17_H_10_N_2_O_3_ (II), has triclinic ($P\overset{®}{1}$) symmetry. The structure displays C–H ⋯ N, C–H ⋯ C hydrogen bonding. In (I), the chlorophenyl and benzoxazole groups adopt a trans configuration with respect to the central cyanomethyle hydrazone moiety. Compound (II) crystallized with two molecules in the asymmetric unit shows cisoid conformation between cyano group and benzoxazole nitrogen, contrary to (I). In (II) the benzodioxole has an envelope conformation (the C17 atom is the flap atom). The molecular geometry obtained using molecular mechanics (MM) calculations has been discussed along with the results of single crystal analysis.

## 1. Introduction 

Benzoxazole derivatives are one of the most important bioactive heterocyclic organic compounds in pharmaceutical chemistry. They have been used as a starting material for synthesis of bioactive structures of pharmaceutical drugs, such as the antibiotic Calcimycin that includes a 2-substituted benzoxazole ring in its molecular structure [1, 2]. Previous studies revealed that substituted benzoxazoles possess diverse chemotherapeutic activities including antibiotic, antimicrobial, antiviral, topoisomerase inhibitors, and antitumor activities [3–6]. Benzoxazoles possess the structural isosteres of natural nucleotides (such as adenine and guanine) which allows them to interact easily with the biopolymers of living systems [7]. Also benzoxazole derivatives have been entered in the synthesis of new classes of antibacterial drug, which has showed activity against bacterial infections [8]. Benzoxazoles are also widely used in industry, such as a photostable highly efficient UV dyes, a dopant in organic light-emitting diodes, chromophores, and chemosensors [9, 10].

It was reported that knowing the crystal structure and conformation of 2-substituted benzoxazole derivatives supports important information for predicting their mode of orientation on the receptor [3]. Then, more bioactive drugs in the pharmaceutical industry could be designed.

In view of the aforementioned literature survey and to support the pharmaceutical and organic chemistry scientists with structural aspects that may be of value in designing new derivatives and potent drugs, we present the geometrical, stereochemical features of two bioactive 2-substituted benzoxazole derivatives comparing their structures with each other and related structures, using X-ray single crystal analysis and molecular mechanics (MM) calculations. The chosen derivatives are 2-[(4-chlorophenylazo) cyanomethyl] benzoxazole, C_15_H_9_ClN_4_O (I) and 2-[(arylidene) cyanomethyl] benzoxazole, C_17_H_10_N_2_O_3_ (II).

## 2. Materials and Methods

### 2.1. Synthesis

**Scheme 1:**
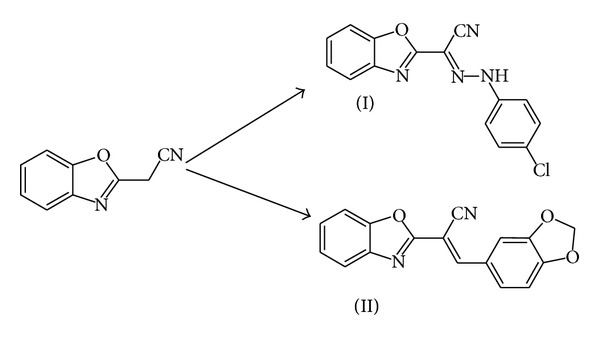
Chemical diagram of the target compounds.

The target compounds have been prepared according to the reported procedure [3] (Scheme 1). They were obtained mainly from diazocoupling of 2-cyanomethyl-benzoxazole with appropriate diazonium acetate to attach hydrazone, cyano, or thiazole, which reported as the function groups of the bioactivity. Melting points were determined in open-glass capillaries on a Gallenkamp melting point apparatus and are uncorrected. The IR spectra were recorded using KBr discs on a Perkin-Elmer 1430 spectrophotometer.

*Compound (I).* An ice-cooled solution of the diazonium acetate [prepared by the addition of solution of sodium nitrite (1 g, 15 mmole) in water (5 mL) to the required arylamine (10 mmole) in acetic acid (10 mL)] was added dropwise with stirring to a solution of 2-cyanomethylbenzoxazole (1.58 g, 10 mmole) in acetic acid (5 mL). Stirring was maintained for 30 minutes after which water was added and the precipitated product was filtered, washed with water, dried, and crystallized by slow evaporation from ethanol. IR of compound (I) (*υ* cm^−1^) is as follows: 3171–3066 (NH); 2226–2223 (C*≡*N); 1611–1599, 1551-1550, 1502–1481 (C=N, NH bending, C=C); 1278–1266, 1097–1087 (C–O–C).

*Compound (II).* Triethylamine (5 drops) and the aldehyde (4 mmole) were added to a stirred solution of 2-cyanomethylbenzoxazole (0.63 g, 4 mmole) in absolute ethanol (10 mL). The reaction mixture was stirred at room temperature for 3 hours during which yellow crystals separated out. The crystalline product was filtered, washed with ethanol, dried, and crystallized by evaporation from dioxane solvent. IR of compound (II) (*υ* cm^−1^) is as follows: 2230–2223 (C*≡*N); 1588–1574, 1513–1502 (C=N, C=C); 1271–1240, 1180–1150, 1040–1022 (C–O–C).

### 2.2. X-Ray Single Crystal Measurements

Crystals were selected and checked for imperfections such as cracks, bubbles, twining, or voids and mounted onto thin glass fibers and glued with epoxy glue. X-ray diffraction data were collected at room temperature on an Enraf-Nonius 590 Kappa CCD single crystal diffractometer with graphite monochromated Mo-K*α* (*λ* = 0.71073 Å) radiation, at National Research Center of Egypt [11, 12]. Crystal data, data collection, and structure refinement details are summarized in Table 1. The relatively large ratio of minimum to maximum corrections applied in the multiscan process (1 nnn) reflects changes in the illuminated volume of the crystal. Changes in illuminated volume were kept to a minimum and were taken into account [13] by the multiscan interframe scaling [14].

The crystal structures were solved using Superflip [15], which revealed the positions of all nonhydrogen atoms and refined by the full matrix least squares refinement based on F² using CRYSTALS package [16]. The anisotropic displacement parameters of all nonhydrogen atoms were refined, and then the hydrogen atoms were all located in a difference map, but those attached to carbon atoms were repositioned geometrically. The H atoms were initially refined with soft restraints on the bond lengths and angles to regularise their geometry (C–H in the range 0.93–0.98, N–H in the range 0.86–0.89 N–H to 0.86 O–H = 0.82 Å) and U_iso_(H) (in the range 1.2–1.5 times U_eq_ of the parent atom). Then, the positions were refined with riding mode (95 Å) [17]. The molecular graphics were prepared using Diamond [18] program.

The crystal data is listed in Table 1. The full crystallographic information can be obtained free of charge using deposit numbers CCDC 675940 and CCDC 692455 for (I) and (II), respectively, via http://www.ccdc.cam.ac.uk/conts/retrieving.html or from the Cambridge Crystallographic Data Centre, Cambridge, UK.

### 2.3. Molecular Mechanics Computations

Molecular mechanics* in vacuo* computations were carried out using HyperChem package [19]. The molecular mechanics (MM+) force field was used as it is developed principally for organic molecules [20–22]. The process of energy minimization was carried out by Steepest Descents method. The conformational energy of the molecule was calculated. The lowest energy conformation is shown and compared to the crystal structures.

## 3. Results and Discussions

### 3.1. Crystal Structure Description

**Figure 1:**
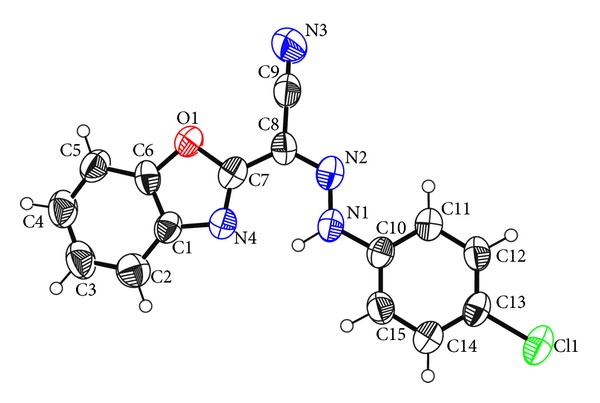
The 50% probability displacement ellipsoids representation of compound (I).

**Figure 2:**
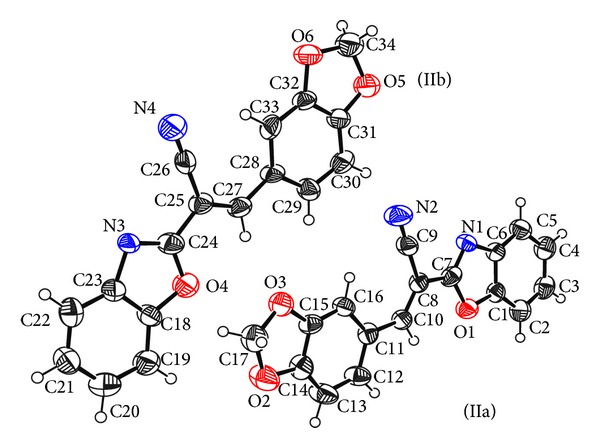
The 50% probability displacement ellipsoids representation of compound (II).

Structures of compounds (I) and (II) consist mainly of benzoxazole connected with different chemical moieties at C7 (Figures 1 and 2). Two independent molecules in the asymmetric unit cell have been found in the second compound, IIa and IIb.

Benzoxazole is almost planar, where the maximum deviation from the mean plane corresponds to the atom C2, −0.013 (3) Å in (I) and the atoms C6, 0.008 (6) Å and O4, −0.012 (4) Å, in (IIa) and (IIb), respectively. This is comparable with the reported structures which have the same moiety, such as 2-(4-aminophenyl)-1, 3-benzoxazole [23], 2-amino-5-chloro-1, 3-benzoxazole [24], and 5-(2-chlorobenzoyl)-1,3-benzoxazol-2(3H)-one [25], also the related structures reported in [26]. The phenyl ring in (I) has planer configuration where the maximum deviation corresponds to the atom C12, 0.010 (3) Å. Benzoxazole group and the phenyl ring adopt a trans configuration with respect to the central cyanomethyle hydrazone moiety, with dihedral angle between the two mean ring planes 180°.

In compound (II), the benzoxazole group is linked to benzodioxol via acrylonitrile moiety. Planar configuration of benzodioxole moiety in (IIb) is confirmed by the deviation of the benzodioxole atoms from their best plane, with maximum deviation at O6, −0.026 (4) Å. However, in (IIa), the dioxole ring adopts the envelope conformation with C17 deviating from the plane defined by the rest of the atoms of the ring (O2-C17) by −0.069 (7) Å. The puckering parameters [27] of this ring are *Q* = 0.109 (6) Å and *φ* = 329 (3)°.

Conformational investigation of the structures reveals that there is cisoid conformation between the cyano group and benzoxazole nitrogen in compound (II) (Figure 2), which in agreement with the reported cisoid conformation of 2-[(3-hydroxy-4-methoxybenzylidene)-cyanomethyl]-benzoxazole [3]. In contrary in compound (I) (Figure 1) the cyano group and benzoxazole nitrogen shows transoid conformation, as reported before such information would add an important way for predicting the geometry of the drug-receptor interaction [3].

**Figure 3:**
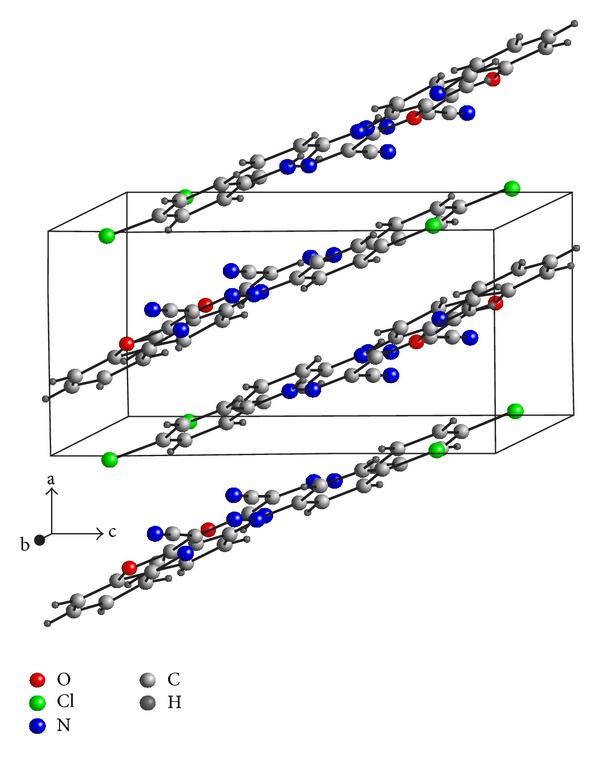
The molecular packing of (I).

**Figure 4:**
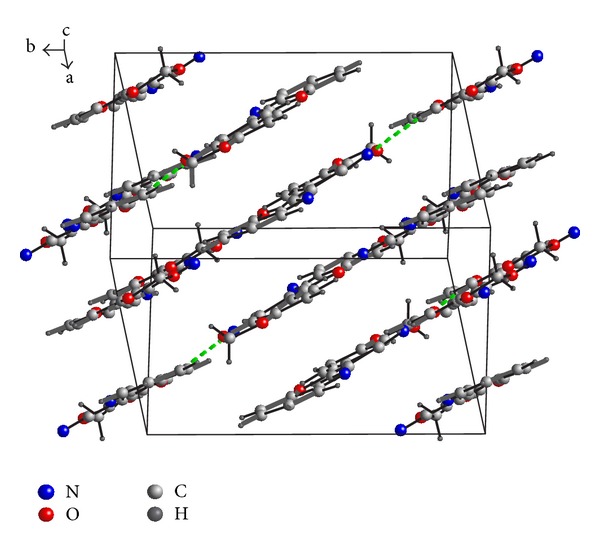
The molecular packing of (II) with the intermolecular interactions shown as dashed line.

The structures are stabilized by the intermolecular interactions and a network of hydrogen bond contacts conformed parallel layers, N-H*⋯*N in compound (I), Table 2, and C–H*⋯*N and C–H*⋯*C in compound (II), Table 3. The packing diagrams of the compounds are shown in Figures 3 and 4.

### 3.2. Molecular Mechanics Computations

**Figure 5:**
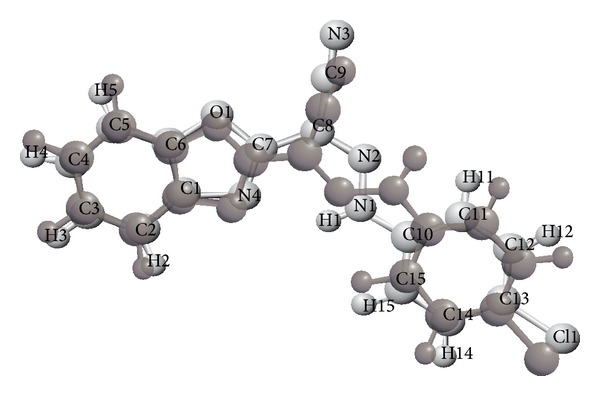
Superimposition view of the calculated structure (black) on the X-ray structure (gray) for the compound (I).

**Figure 6:**
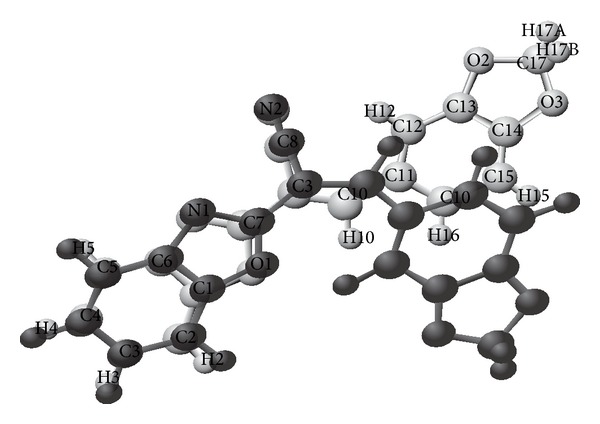
Superimposition view of the calculated structure (black) on the X-ray structure (gray) for the compound (II).

The minimum energy structure obtained by molecular mechanics of the investigated compounds did not match well the crystal structures obtained experimentally, Figures 5 and 6. However, trans configuration between benzoxazole group and the benzene ring with respect to the central cyanomethyle hydrazone moiety appears also in the theoretical structure. The global energy minimum conformations as calculated by molecular mechanics* in vacuo* in agreement with the above-mentioned crystallographically observed conformations, where cisoid conformation has noticed only in (II).

Tables 4 and 5 show selected geometrical values of experimentally obtained structure using X-ray (Exp.) and molecular mechanics (MM) for (I) and (II), respectively. The bonds of the benzoxazole ring obtained theoretically in (I) and (II) almost agree with those obtained experimentally with X-ray diffraction. On the other hand, in (I) the deference is 180° degree in N2–N1–C10–C15 and H11–N1–C10–C11 torsion angles. Also, there is considerable variation of C11–C10–C8–C7 torsion angle in (II). It was found that benzodioxole ring has orientation in the experimental structure different from the orientation of the same group in the theoretical structure.

However, the energy of the experimental structures was higher than the energy of the structure obtained using molecular mechanics by the values 5.8 kcal·mol^−1^ in compound (I) and 1.9 kcal·mol^−1^ in compound (II). This variation may be due to the fact that the experimental structure of the investigated compounds in crystal conditions (i.e., the neighbouring molecules, hydrogen bonding, and other nonbonded interactions in the crystal lattice environment) is taken into account. This is in agreement with what was reported in the literature showing that the effects of hydrogen-bonding and van der Waals interactions in the crystal structure cause the molecules to adopt higher-energy conformations, which correspond to local minima in the molecular potential energy surface [28]. This result in consent with the reported notation, which states that the crystallographically observed molecular architecture is a local energy minimum in the absence of its crystal lattice environment [29].

## 4. Conclusions

Crystallographic and stereochemical study of 2-substituted benzoxazole derivatives, 2-[(4-chlorophenylazo) cyanomethyl] benzoxazole and 2-[(arylidene) cyanomethyl] benzoxazole, has been introduced using X-ray single crystal and MM. The study has reported that the crystal structures of the two compounds have a triclinic ($P\overset{-}{1}$) space group. The study showed in (II) that cisoid conformation between the cyano group and benzoxazole nitrogen and the benzodioxole has an envelope conformation. The features of the whole molecules obtained using MM do not match well those obtained by X-ray; however, the results have supported the conformation discussion.

## Supplementary Material

Full crystal structure data of 2-[(4-chlorophenylazo) cyanomethyle] benzoxazole, (I), and 2-[(Arylidene) cyanomethyl] benzoxazoles, (II), are presented in the standard Crystallographic Information File (CIF).

## Figures and Tables

**Table 1:** Crystal data of the studied compounds.

	(I)	(II)
Crystal data		
Chemical formula	C_15_H_9_ClN_4_O	C_17_H_10_N_2_O_3_
*M*_*r*_	296.72	290.28
Crystal system, space group	Triclinic, $P\overset{-}{1}$	Triclinic, $P\overset{-}{1}$
Temperature (K)	298	298
*a*, *b*, *c* (Å)	7.5050 (7), 7.4836 (10), 13.4301 (17)	7.4919 (5), 13.0828 (9), 14.1914 (14)
*α*, *β*, *γ* (°)	106.488 (6), 90.485 (7), 102.759 (8)	94.355 (3), 101.180 (3), 102.504 (6)
*V* (Å^3^)	703.37 (15)	1322.07 (19)
*Z*	2	4
Radiation type	Mo *Κα*	Mo *Κα*
*µ* (mm^−1^)	0.28	0.10
Crystal size (mm)	0.12 × 0.10 × 0.09	0.12 × 0.11 × 0.08

Data collection		
Absorption correction	Multiscan	Multiscan
*T*_min⁡_, *T*_max⁡_	0.97, 0.98	0.99, 0.99
Number of measured, independent and observed [*I* > 2.0*σ*(*I*)] reflections	4166, 3007, 1543	7240, 4861, 2163
*R*_int⁡_	0.031	0.084
(sin⁡⁡*θ*/*λ*)_max⁡_ (Å^−1^)	0.655	0.617

Refinement		
*R*[*F* ^2^ > 2*σ*(*F* ^2^)], *wR*(*F* ^2^), *S*	0.071, 0.070, 1.13	0.089, 0.155, 0.97
Number of reflections, parameters, and restraints	1309, 64, 0	1738, 133, 0
Δ*ρ*_max⁡_, Δ*ρ*_min⁡_ (*e* Å^−3^)	0.25, −0.26	0.33, −0.25

**Table 2:** Hydrogen-bond geometry (Å, °) for (I).

D–H*⋯*A	*D*–H	H*⋯*A	D*⋯*A	D–H*⋯*A
N1–H11*⋯*N4	0.950	1.975	2.710 (4)	133

**Table 3:** Hydrogen-bond geometry (Å, °) for (II).

D–H*⋯*A	D–H	H*⋯*A	D*⋯*A	D–H*⋯*A
C13–H131*⋯*N4^i^	0.950	2.519	3.431 (8)	161
C16–H161*⋯*N2	0.950	2.600	3.450 (8)	149
C16–H161*⋯*C9	0.950	2.433	3.063 (8)	124
C30–H301*⋯*N2	0.950	2.574	3.464 (8)	156
C33–H331*⋯*C26	0.950	2.426	3.052 (8)	123

Symmetry code: ^i^ *x* − 1, *y* − 1, *z*.

**Table 4:** Selected geometrical values of molecular mechanics and experimentally obtained structures of compound (I).

Bond length (Å)	Exp.	MM	Bond angles (°)	Exp.	MM
N1–N2	1.317 (3)	1.354	C1–C2–C3	117.1 (3)	117.07
N2–C8	1.307 (4)	1.3485	C3–C4–C5	121.6 (3)	121.17
C8–C9	1.434 (4)	1.31	O1–C7–N4	115.7 (3)	115.84
C1–C2	1.381 (4)	1.390	Cl1–C13–C14	119.6 (3)	119.9
C6–C1	1.387 (4)	1.384	C6–O1–C7–C8	179.5 (4)	180
Cl1–C13	1.737 (3)	1.726	C10–N1–N2–C8	179.1 (5)	179.99
C10–C11	1.375 (4)	1.398	C7–C8–C9–N3	130 (2)	180
C11–C12	1.395 (4)	1.398	C5–C6–C1–N4	179.6 (5)	180
C13–C12	1.369 (5)	1.396	N2–N1–C10–C15	179.9 (5)	0
C13–C14	1.389 (5)	1.396	H1–N1–C10–C11	177.5 (8)	0
N4–C1	1.400 (4)	1.348	H1–N1–C10–C15	1.0 (8)	180
N4–C7	1.294 (4)	1.358	C10–N1–H1–N4	179.7 (13)	180

**Table 5:** Selected geometrical values of molecular mechanics and experimentally obtained structures of compound (II).

Bond length (Å)	Exp.	MM	Bond angles (°)	Exp.	MM
N1–C6	1.403 (5)	1.348	C1–C6–C5	120.7 (4)	121.59
N1–C7	1.279 (5)	1.363	C9–C8–C7	112.1 (4)	112.90
C6–C5	1.366 (5)	1.390	C8–C10–C11	131.3 (4)	221.59
C6–C1	1.378 (5)	1.381	C13–C14–C15	123.1 (5)	122.58
C1–C2	1.372 (6)	1.390	O2–C17–O3	107.6 (3)	105.35
C5–C4	1.373 (6)	1.399	C13–C12–C11	117.6 (4)	116.18
C4–C3	1.395 (6)	1.403	C7–O1–C1–C2	179.8 (9)	180
N2–C9	1.132 (1)	1.15	O1–C1–C2–C3	179.8 (11)	180
C9–C8	1.423 (6)	1.321	C7–N1–C6–C5	179.0 (10)	180
C7–C8	1.460 (5)	1.345	N1–C7–C8–C9	4.2 (6)	0
C11–C12	1.419 (5)	1.417	C11–C10–C8–C7	178.1 (11)	0
C10–C11	1.446 (5)	1.353	C13–C12–C11–C10	−179.2 (10)	179.99
