# Supplementary material for: Crystal Structure and Stereochemistry Study of 2-Substituted Benzoxazole Derivatives
Source: ISRN Org Chem. 2014 May 13;2014:728343. doi: 10.1155/2014/728343 (PMC4040200; doi:10.1155/2014/728343)
Supplement: Supplementary file 1 — Full crystal structure data of 2-[(4-chlorophenylazo) cyanomethyle] benzoxazole, (I), and 2-[(Arylidene) cyanomethyl] benzoxazoles, (II), are presented in the standard Crystallographic Information File (CIF). [file 728343.f1.zip › 728343_sup-mabied.doc]

Full crystal structure data of 2-[(4-chlorophenylazo) cyanomethyle] benzoxazole, (I), and 2-[(Arylidene) cyanomethyl] benzoxazoles, (II), are presented in the standard Crystallographic Information File (CIF).
